# Deciphering community interactions of sulfate-reducing microorganisms in complex microbial communities of marine sediments

**DOI:** 10.1128/mbio.00513-23

**Published:** 2023-06-28

**Authors:** Kenneth Wasmund

**Affiliations:** 1 School of Biological Sciences, University of Portsmouth, Portsmouth, United Kingdom; University of California, Irvine, Irvine, California, USA

**Keywords:** sulfate-reducers, marine sediments, ecological networks

## Abstract

Sulfate-reducing microorganisms (SRM) are key players in global sulfur and carbon cycles, especially in anoxic marine sediments. They are critical in anaerobic food webs because they consume fermentation products like volatile fatty acids (VFAs) and/or hydrogen produced from other microbes that degrade organic matter. Apart from this, the interplay between SRM and other coexisting microorganisms is poorly understood. A recent study by Liang et al. provides intriguing new insights about how the activity of SRM influence microbial communities. Using an elegant combination of microcosm experiments, community ecology, genomics, and *in vitro* studies, they provide evidence that SRM are central in ecological networks and community assembly, and interestingly, that the control of pH by SRM activity has a substantial impact on other key bacteria, like members of the Marinilabiliales (Bacteroidota). This work has important implications for understanding how marine sediment microbes function together to provide important ecosystem services like recycling organic matter.

## TEXT

Sulfate-reducing microorganisms (SRM) are a functional guild of microbes that use sulfate as a terminal electron acceptor for anaerobic respiration. They are especially key among the diverse and massive populations of microbial life that inhabit the vast expanses of marine sediments underlying our oceans (1). This is mainly because large expanses of marine sediments are anoxic, and marine waters are rich in sulfate (SO_4_^2-^), which diffuses into the sediments to supply SRM. This living and breathing marine mud, or the “guts of our oceans,” plays a critical role in global element cycles because large amounts of organic material are deposited there. This organic material is either degraded and recycled by microbes back to CO_2_ or buried into the deep subsurface and therefore locked away from the carbon cycle (2). Therefore, the activity of sedimentary microbes, including SRM, plays pivotal roles in global carbon cycles. Understanding how SRM interact with other microbes in our global sedimentary bioreactor is therefore crucial for understanding how the marine carbon cycle works.

The anaerobic degradation of organic material in marine sediments is intriguing because complex microbial communities work together to hydrolyze, ferment, and dispose of fermentation by-products like volatile fatty acids (VFAs) and/or hydrogen (3). For this, distinct functional guilds of microbes take specialized roles in the different steps of the anaerobic food web. SRM are well known as key players at the “back-end” of this anaerobic food web, i.e., they are fundamental to the terminal oxidation of VFAs and/or hydrogen, which is critical for the complete mineralization of organic material. In fact, about 50% of all organic matter degradation is facilitated by SRM in organic-rich coastal and continental shelf sediments (4). Thus, SRM are key players in global carbon cycles, not just sulfur cycles. While SRM are important, they coexist with other key members, such as primary hydrolyzers of macromolecular organic matter (5, 6). SRM therefore likely have strong ecological interactions with other microbes, yet these remain poorly understood.

In a recent *mBio* study by Liang et al., the authors aimed to shed new light on how SRM interact with and exert control over other key members of a marine sediment microbial community (7). In brief, they found that SRM indeed have a major effect on community ecology and explore new dimensions regarding their interactions with other community members.

The crux of the work was based on a series of highly replicated anaerobic marine sediment microcosms, whereby half were treated with molybdate, a structural analogue of sulfate, and thus, a specific inhibitor of SRM (8). These inhibited treatments are powerful because they allow us to specifically examine the effects of SRM activity on the ecological dynamics, assembly, and interaction networks of SRM within the communities, while also examining the resulting biogeochemical effects of blocking SRM activity in the systems. Control microcosms without molybdate were run in parallel, to mimic natural communities where SRM retain their “normal” community interactions and biogeochemical activities. The authors allowed the microcosms to incubate for about 1 month and followed the ecological dynamics of the microbial communities by high-throughput amplicon sequencing of 16S rRNA genes and advanced ecological analyses of the data, in addition to key biogeochemical measurements such as concentrations of sulfur compounds, VFAs, inorganic carbon, and importantly, pH.

The biogeochemical measurements showed what we might expect when SRM are inhibited, i.e., no removal of sulfate, indicating the SRM were indeed blocked; accumulations of various VFAs, indicating the SRM have stopped using them but fermenters were still active; and interestingly, a significant drop in pH probably caused by the acidic accumulating VFAs. Thus, the important role of SRM in the biogeochemical functioning of the sediment community was confirmed.

When examining the ecological dynamics of the community, Liang et al. explored various quantitative ecological measures to show that SRM are important members of central ecological networks; most importantly, they appear to be involved in an array of positive interactions in ecological networks. In stark contrast, in treatments where SRM were inhibited, the networks broke down, were more stochastic, and “looser” associations among community members occurred. Together, these analyses highlight how SRM are integral to community interactions and community stability and could thus also be inferred to be critical for biogeochemical functioning of the whole communities. It also nicely shows how the communities are highly adapted to working together in ecological networks to perform the key ecosystem function of recycling organic material anaerobically.

When looking at the community sequence data, the authors also noticed that the molybdate additions that are only supposed to inhibit SRM, also reduced the abundances and disrupted network interactions of taxa from several other families, with one such group being members of the Marinilabiliales (Bacteriodota). This group of bacteria was interesting because they were also found to be tightly intertwined within the ecological networks of key SRM in the control incubations, suggesting they interact under normal conditions. This was also intriguing because most of these other non-SRM don’t have enzymes that would be affected by molybdate, indicating something else might be strongly affecting their ecology and niche.

To hone in on a reason, the authors suspected the pH effect. To explore this, they isolated a collection of Marinilabiliales strains from the sediments and tested them *in vitro* for pH sensitivity. Importantly, they first showed that the strains are not inhibited by molybdate at concentrations used in the microcosm experiments, suggesting they were indeed likely affected by something else. When testing their pH preferences *in vitro*, it was apparent that this group of organisms had reduced growth at weakly acidic pH levels seen in the sediment microcosms when SRM were inhibited, i.e., around pH 6.4. This coincided with this pH level being very close to the minimal pH tolerance of the strains. Thus, it seems the SRM are critical for maintaining the pH niche of these taxa in marine sediments.

In a nice follow-up experiment, the authors repeated the microcosm incubations, but added a buffering agent (HEPES) to the SRB-inhibited treatments. This kept the pH up around 7.4 even though VFAs would have accumulated, and thus kept the pH similar to the sediments of the control microcosms where SRB consume the VFAs efficiently. Sequencing of the community then showed that members of the family Marinifilaceae (a family of the Marinilabiliales) were less affected when the sediments were buffered, further supporting that pH was specifically affecting their abundances in the communities.

Finally, the authors postulated that further metabolic interactions based on metabolite exchanges of key metabolites could also help explain the interaction between SRM and Marinilabiliales. To explore this, the authors performed genomic analyses of various taxa from the sediments, in order to identify genetic potential for biosynthetic capabilities, or lack thereof. While various auxotrophies were identified, several complementing metabolite biosynthetic capabilities were found for SRM and Marinilabiliales, e.g., L-proline, L-ornithine, L-cysteine, and vitamins B7 and B12, suggesting exchange of these could promote their growth and interactions. The authors then also went back to the lab and showed that supplementing these specific metabolites to 10 Marinilabiliales strains indeed facilitated better growth in the minimal media used. This therefore suggested SRM may fill gaps in some of the auxotrophies of Marinilabiliales. Finally, the authors also point out that other factors like the hydrogen sulfide produced by SRM may be another key factor shaping the communities.

Overall, this work provides new and unique perspectives on how interlinked anaerobic microbial communities function and how SRM alter the niche space of accompanying community members. While teasing apart such factors can be difficult in complex environmental samples, the authors did a commendable job of combining experimental microbial community ecology, with complementary *in vitro* and *in silico* studies. Such approaches will undoubtedly be useful for deciphering microbial community ecology and functioning in other contexts in the future too.
